# DYRK1A roles in human neural progenitors

**DOI:** 10.3389/fnins.2025.1533253

**Published:** 2025-03-13

**Authors:** Jeremie Courraud, Angélique Quartier, Nathalie Drouot, Irene Zapata-Bodalo, Johan Gilet, Alexandra Benchoua, Jean-Louis Mandel, Amélie Piton

**Affiliations:** 1 Institut de Génétique et de Biologie Moléculaire et Cellulaire, Illkirch, France; 2 Centre National de la Recherche Scientifique, UMR7104, Illkirch, France; 3 Institut National de la Santé et de la Recherche Médicale, U964, Illkirch, France; 4 Strasbourg University, Illkirch, France; 5 I-Stem, Evry, France; 6 Genetic Diagnosis Laboratory, Strasbourg University Hospital, Strasbourg, France; 7 Institut Universitaire de France, Paris, France

**Keywords:** DYRK1A, human neural stem cells, interactome, gene expression, cell proliferation, RNF114, p21/CDKN1A, neurodevelopmental disorders

## Abstract

**Introduction:**

Mutations in *dual-specificity tyrosine phosphorylation-regulated kinase 1A* (DYRK1A) represent one of the most prevalent monogenic causes of neurodevelopmental disorders (NDDs), often associated with intellectual developmental disorder and autism spectrum disorder. DYRK1A encodes a dual-specificity kinase (tyrosine and serine/threonine) that plays a key role in various cellular processes and is a critical regulator of nervous system development.

**Methods:**

For the first time, we have characterized the DYRK1A interactome and study the consequences of DYRK1A depletion in human neural stem cells (hNSCs).

**Results:**

We identified 35 protein partners of DYRK1A involved in essential pathways such as cell cycle regulation and DNA repair. Notably, five of these interactors are components of the anaphase-promoting complex (APC), and one is an additional ubiquitin ligase, RNF114 (also known as ZNF313), which is known to target p21. Many of these identified partners are also linked to other human NDDs, and several others (e.g., DCAF7 and GSPT1) may represent novel candidate genes for NDDs. DYRK1A knockdown (KD) in hNSCs using siRNA revealed changes in the expression of genes encoding proteins involved in extracellular matrix composition and calcium binding (e.g., collagens, TGFβ2 and UNC13A). While the majority of genes were downregulated following DYRK1A depletion, we observed an upregulation of early growth factors (EGR1 and EGR3), as well as E2F2 and its downstream targets. In addition, DYRK1A-KD led to a reduction in p21 protein levels, despite an increase in the expression of a minor transcript variant for this gene, and a decrease in ERK pathway activation.

**Discussion:**

Together, the DYRK1A interactome in hNSCs and the gene expression changes induced by its depletion highlight the significant role of DYRK1A in regulating hNSC proliferation. Although the effects on various growth signaling pathways may appear contradictory, the overall impact is a marked reduction in hNSC proliferation. This research underscores the pivotal role of DYRK1A in neurodevelopment and identifies, among DYRK1A’s protein partners and differentially expressed genes, potential novel candidate genes for NDDs and promising therapeutic targets for DYRK1A syndrome.

## Introduction

Intellectual developmental disorder (IDD) and autism spectrum disorder (ASD) are two groups of neurodevelopmental disorders (NDDs) that share a significant genetic contribution and exhibit strong overlap both at the clinical and genetic levels. Single genetic events account for an important part of IDD cases and a non-negligible part of ASD cases. More than a thousand genes have been implicated in monogenic forms of IDD or ASD. For the majority of them, little is known about the pathophysiological mechanisms leading to IDD and ASD. Some genes are more frequently mutated than others, such as *dual-specificity tyrosine phosphorylation-regulated kinase 1A* (*DYRK1A*; Gonzalez-Mantilla et al., 2016). The first disruptions of *DYRK1A* were identified in individuals with intrauterine growth retardation, primary microcephaly, and epilepsy (Møller et al., 2008). The clinical spectrum associated with heterozygote mutations in *DYRK1A* was further refined with the publication of additional patient cases. This spectrum includes IDD, feeding difficulties, poor or absent language, microcephaly, autistic traits, epilepsy, and a typical facial gestalt (Courraud et al., 2021; van Bon et al., 2016; Bronicki et al., 2015). Loss-of-function mutations were also identified in cohorts of ASD individuals (O’Roak et al., 2012; Iossifov et al., 2014), but all have IDD. Interestingly, an increased dosage of *DYRK1A*, located on chromosome 21, is thought to participate in the cognitive manifestations of Down syndrome (Altafaj et al., 2001), suggesting that a correct balance of *DYRK1A* dosage is essential for brain development and cognitive function.

The *DYRK1A* gene codes a dual tyrosine-serine/threonine (Tyr-Ser/Thr) kinase protein that belongs to the DYRKs kinase family. Its main isoform comprises 753 amino acids (Becker, 2011). DYRK1A has the general structure of the DYRKs kinase family, including a DYRK Homology-box (DH) domain, two Nuclear Localization Signal sequences (NLS), and a central catalytic domain. The catalytic domain contains Tyrosine 321, which is involved in the activation of DYRK1A through autophosphorylation. Additionally, DYRK1A features a leucine zipper (bZIP) domain, suggesting it can form dimers or multimers with other nuclear proteins, such as transcription. It also includes Ser/Thr repeats that facilitate interaction with target proteins, a poly-histidine sequence that directs it to nuclear speckle compartments, and a PEST domain essential for its degradation and precise regulation of its cellular concentration. DYRK1A is ubiquitously expressed during embryonic development and in adults. Its location is both cytoplasmic and nuclear depending on the cell type and stage of development (Hämmerle et al., 2008). By the number and the diversity of its protein targets, DYRK1A regulates numerous cellular functions (Tejedor and Hämmerle, 2011; Duchon and Herault, 2016). DYRK1A regulates cytoskeleton-associated proteins, such as TAU, MAP1B, *β*-tubulin, and others, and has been shown to play a role in the regulation of dendritic morphogenesis in rodents and *Drosophila* (Ori-McKenney et al., 2016; Ryoo et al., 2007). DYRK1A modulates synaptic plasticity by regulating NMDA receptor expression at the membrane surface (Grau et al., 2014). DYRK1A interacts with proteins involved in endocytosis such as Dynamin1a or Amphiphysin1 (Murakami et al., 2006) and interacts with the light chain of Clathrin and Endophilin A1 (Murakami et al., 2009; Huang et al., 2004). DYRK1A phosphorylates several proteins involved in cell cycle regulation, such as cyclin D1 or p27^Kip1^, and therefore is involved in the regulation of mouse neural progenitor proliferation (Hämmerle et al., 2008). In addition to its cytoplasmic targets, DYRK1A also regulates numerous nuclear proteins: It interacts with and phosphorylates transcription factors such as acetyltransferases CBP and p300 (Li et al., 2018) and binds the chromatin remodeling SWI/SNIF complex (Lepagnol-Bestel et al., 2009), Histone H3 (Jang et al., 2014), and RNA polymerase type II (Di Vona et al., 2015). On the one hand, DYRK1A positively regulates CREB (Yang et al., 2001) and GLI1 (Ehe et al., 2017) activity, and the nuclear translocation of NFAT (Gwack et al., 2006) and, on the other hand, negatively regulates REST complex stability (Lu et al., 2011). We recently demonstrated that loss-of-function variants in *DYRK1A* induce specific changes in DNA methylation in blood, consistent with a role in chromatin remodeling (Courraud et al., 2021). Other nuclear substrates of DYRK1A have been reported, including splicing factors (Shi et al., 2008).

Although numerous studies have unraveled some of the functions of DYRK1A, the majority of the work about its role in the brain has been performed in mice (Fotaki et al., 2002; Fotaki et al., 2004), *Drosophila* (Fischbach and Heisenberg, 1981; Tejedor et al., 1995), or, more recently, in *Xenopus* models (Blackburn et al., 2019; Willsey et al., 2020). Therefore, little is known about its role in human neural cells and about which of its cellular functions are critical for early human brain development and functioning. To characterize the role of DYRK1A in human neuronal progenitors, we performed proteomic studies to identify its interactome as well as transcriptomic analysis to identify changes in gene expression occurring after its knockdown (KD). We also analyzed the effect of *DYRK1A*-KD on hNSC ability to proliferate.

## Materials and methods

### Cell culture, transfection, and proliferation assay

Human neuronal stem cells (hNSCs) from two genetic backgrounds (SA001/hNSC_1_ and GMO1869/hNSC_2_) were obtained from I-Stem and are described in previous studies (Boissart et al., 2012; Courraud et al., 2023). hNSCs were seeded on poly-ornithine-and laminin-coated dishes and maintained in N2B27 medium (DMEM/F12 and Neurobasal medium [1:1] supplemented with N2, B27, 2-mercaptoethanol [all from Invitrogen]), BDNF (20 ng/mL), FGF-2 (10 ng/mL; both from PeproTech), and EGF (R&D Systems; 10 ng/mL). Culture and quality controls of hNSCs were performed as described in Quartier et al. (2018). hNSCs were transfected using INTERFERin reverse transfection protocol (Polyplus-transfection), with *Scramble* siRNA, a pool of *DYRK1A* siRNA (120 nM final concentration), or the transfecting agent only. Cells were harvested 48 h after transfection for RNA and protein extractions. All cell lines were grown at 37°C in 5% CO_2_. For proliferation assay, reverse transfection of hNSCs (20,000 cells/cm2) was performed in 96-well plates using INTERFERin reagent and siRNA according to the manufacturer’s recommendations. Each day, a plate was fixed and stained with DAPI, and the number of nuclei in each condition was counted using CellInsight automated microscope and HCS Studio software (Thermo Fisher Scientific).

### Immunoprecipitation coupled to mass spectrometry

Proteins extracted from untreated hNSC_1_ were immunoprecipitated with DYRK1A antibodies targeting the N-terminal and C-terminal regions of the protein, respectively (Cohesion Biosciences #CPA1357, Immunogen sequence aa 39-51 and Abnova H00001859-M01, aa 674–763). As negative controls, we used free beads without antibodies as well as mouse anti-rabbit (MAR, 211–002-171, Jackson ImmunoResearch) and rabbit anti-mouse (RAM, 315–005-044, Jackson ImmunoResearch) antibodies as described (Mattioli et al., 2019). Immunoprecipitations were validated using Western blot as previously described (Mattioli et al., 2019) before the mass spectrometry analyses (Proteomic platform, IGBMC). In brief, the samples were treated with LysC/trypsin for digestion and injected into Orbitrap ELITE/C18 Accucore 50 cm (20 μL 0.1%TFA/1 μL) for 2 h runs in triplicate. Data were processed using Proteome Discoverer 2.2 software with Homosapiens_190716_reviewed. fasta and contaminants_190528.fasta databases. Thresholds were set at 1% FDR with a minimum of two unique peptides per protein. To consider a protein as a candidate interactor, we applied the thresholds to keep only proteins with (1) peptide-spectrum matching (PSM) sum of any of the control conditions (Beads, MAR, GAR) inferior to 5, (2) a sum of PSM for the three replicates of the test conditions (DYRK1A C-term or DYRK1A N-term) greater or equal to 5 with a positive value for each replicate, and (3) a ratio of NSAF between anti-DYRK1A and control conditions ≥1.5. Enrichment analysis was realized using Database for Annotation, Visualization, and Integrated Discovery (DAVID) using proteins detected in the mass spectrometry experiments (minimum of two peptides per protein) as background. The list of already known DYRK1A interactors was obtained from BioGRID and from previously published proteomic studies (Varjosalo et al., 2013; Li et al., 2015; Menon et al., 2019; Roewenstrunk et al., 2019; Guard et al., 2019; Huttlin et al., 2021). Network analysis was performed using Poteo3Dnet.^1^ Information about pathologies caused by mutations in genes encoding DYRK1A interactors was retrieved using Human Phenotype Ontology. The probability of the genes being intolerant to the loss-of-function variant was obtained from GnomAD (pLI). Gene expression profiles during brain development were obtained from BrainSpan data and were correlated with the *DYRK1A* expression profile (Pearson’s correlation).

### Western blot

Cells were lysed in RIPA buffer (50 mM Tris–HCl pH 7.5, 150 mM NaCl, 0.25% sodium deoxycholate, 1% NP-40) supplemented with protease inhibitor cocktail and phosphatase inhibitor cocktail. A total of 5 to 50 μg of protein lysate was separated on 10% SDS-PAGE and transferred to a polyvinylidene fluoride (PVDF) membrane. Membranes were blocked in 5% non-fat dry milk diluted in tris-buffered saline with tween 20 (50 mM Tris, 150 mM NaCl, 0.05% Tween 20) and probed using the antibodies overnight at 4°C. GAPDH was used as a loading control. Incubation with appropriate HRP-conjugated secondary antibody was followed by detection using Immobilon Western Chemiluminescent HRP Substrate (Merck Millipore, Darmstadt, Germany). The antibodies used were anti-GPADH (G9545 Sigma Aldrich), anti-DYRK1A N-ter (CPA1357 Cohesion Biosciences), anti-DYRK1A C-ter (H00001859-M01 Abnova), pan ERK1/2 (4696S Cell signaling technology), p21/CDKN1A (2947S Cell signaling technology), PTBP2 (H00058155-M01 VWR International), RNF114 (Fisher Scientific, PA5-112261), phospho ERK1/2 (9,101 Cell signaling technology), pan mTOR (2,972 Cell signaling technology), phospho mTOR (2,974 Cell signaling technology), pan PKC (P5704 clone MC5 Sigma), and phospho PKCα (sc-208 Santa Cruz).

### RNA sequencing and RT-qPCR

RNAs were extracted from two series (biological replicates) of hNSC_1_ treated with siRNA targeting *DYRK1A*, *Scramble* siRNA, or transfection agent alone for 48 h and sequenced as previously described (Mattioli et al., 2020). DESeq was used to detect changes in gene expression, and splicing changes were identified using LeafCutter. Genes with a change in mRNA level with an adjusted *p*-value of <0.1 were considered differentially expressed genes (DEG). Gene ontology enrichment was performed using DAVID tools on all the DEG using all the genes expressed in hNSCs as background (number of reads normalized and divided by the median of transcripts length in kb >50). A list of E2F and TGFB1 targets was retrieved from the MSigDB Molecular Signatures Database.^2^ Expression changes were confirmed for the most significant DEG in a third series of hNSC_1_ and in three series (biological replicates) of hNSC_2_: After RNA isolation, reverse transcription reactions (Invitrogen SuperScript IV) were performed using 200–500 ng RNA, followed by qPCR on cDNA on LightCycler 480 II (Roche) using the QuantiTect SYBR Green PCR Master Mix (Qiagen) or RT-qPCR multiplex using 48*48 array Fluidigm Biomark technology (GenomEast platform). A list of primers is available upon request.

### SA-*β* galactosidase assay and cell irradiation

Cells were fixed 4 days after siRNA transfections with 0.5% glutaraldehyde solution for 15 min at room temperature and washed twice with PBS/MgCl_2_ pH 5.95. Fixed cells were incubated with freshly prepared X-gal staining solution (1 mg/mL X-gal), 5 mM potassium ferrocyanide, and 5 mM potassium ferricyanide in PBS/MgCl_2_ pH 6 at 37°C for 20 to 48 h. Cells were washed three times with H_2_O and pictured in a bright field (20X magnification). Counting was done manually using the Cell Counter plugin in FIJI software, and 1,400 to 3,200 cells were counted for each condition. As a positive control of SA-β galactosidase labeling, a batch of untreated cells was irradiated at 8Gy using the “CellRad System Precision,” then fixed and labeled as previously described.

## Results

### Characterization of DYRK1A interactome in human neural stem cells

**Figure 1:**
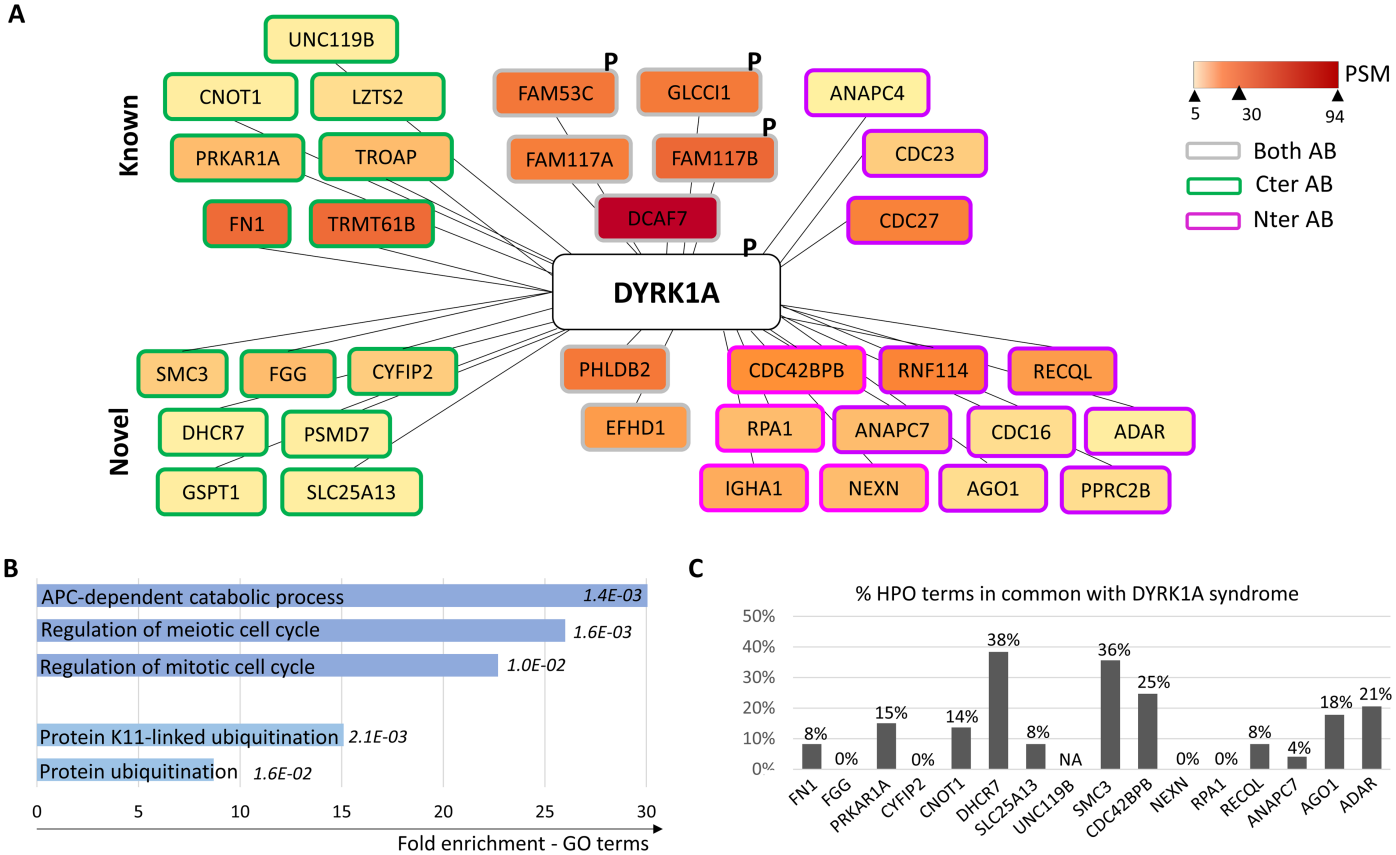
Representation of DYRK1A protein interactome in human neural stem cells. **(A)** Protein interactome of DYRK1A: boxes are filled with a yellow to dark red gradient corresponding to the number of peptide-spectrum matching (PSM) values (see Supplementary Table S1); proteins found using the anti-DYRK1A antibody directed against the C-terminal part of the protein are circled in green, those found using the one directed against the N-terminal part in pink, and those found with both antibodies in gray; “P” indicates that phosphorylation events have been identified. **(B)** Significant enrichment in GO terms among DYRK1A interactors. **(C)** Number of Human Phenotype Ontology (HPO) terms of DYRK1A syndrome reported in the diseases associated with DYRK1A’s partners.

DYRK1A interactome has been studied in different cell types, including HeLa, HEK293T, human glioblastoma T98G, and human neuroblastoma SH-SY-5Y cell lines (Varjosalo et al., 2013; Li et al., 2015; Menon et al., 2019; Roewenstrunk et al., 2019; Guard et al., 2019; Huttlin et al., 2021), which led to a list of 552 interactors (BioGRID). To identify protein partners of DYRK1A in human neural stem cells (hNSC_1_), we performed immunoprecipitations on endogenous DYRK1A using antibodies recognizing either the C-terminal (Cter) or the N-terminal (Nter) parts of the protein. Mass spectrometry analysis of immunoprecipitated proteins (IP-MS) revealed, in addition to DYRK1A, 35 protein interactors with 7 detected with both antibodies. Among them, 15 had previously been identified as DYRK1A partners in other cell types in the literature, and 20 were novel interactors (Table 1; Figure 1A). Phosphorylation analysis on IP-MS data identified phosphorylation events on DYRK1A (Tyr321) and in three partners: FAM117B (Ser106), GLCCI1 (Ser30 and Ser303), and FAM53C (Ser324). The partners identified were significantly enriched in proteins involved in the regulation of cell cycle and protein ubiquitination (Figure 1B), such as for instance members of the anaphase-promoting complex (APC) but also RNF114, alias ZNF313, a zinc-finger E3 ligase involved in cell cycle progression and senescence repression known to ubiquitinate p21/CDKN1A. Among the five members of the APC identified as DYRK1A’s partners in hNSC, three (CDC27/ANAPC3, ANAPC4, and CDC23/ANAPC8) had already been identified in previous studies (Lepagnol-Bestel et al., 2009; Li et al., 2018) but two (CDC16/ANAPC6 and ANAPC7) had not previously been described as DYRK1A interactors (Figure 1A; Supplementary Figure S1). Given that all these proteins are components of the APC complex, immunoprecipitation likely captures the entire complex rather than reflecting direct interactions between DYRK1A and each subunit. Apart from APC, additional clusters of interactions of proteins involved in DNA repair, mRNA regulation at the transcriptional and posttranscriptional level, and intracellular signaling pathways were also identified (Supplementary Figure S1).

**Table 1:** List of DYRK1A interactors identified by Mass-Spectrometry in human neural stem cells

**Protein** **Name**	**PSM number**		**Phospho**	**Known partner**	**Involvement in human disease**	**Intolerance to LoF (pLI) [0-1]**	**Correl. Expression** **[-1-+1]**
	**C ter**	**Nter**					
DYRK1A	97	80	Y321	NA	NDD^1^	1	1
DCAF7	56	38		yes^a,c,d,e,f^	NA	0.93	0.81
FAM117B	21	12	S106	yes^a,c,d,f^	NA	0.01	0.80
FAM53C	13	11	S324	Yes^a,c,d,e,f^	NA	0.07	0.12
PHLDB2	8	15		-	NA	0	-0.31
GLCCI1	16	6	S30, S303	yes^a,b,c,d,e^	NA	0.06	0.63
FAM117A	15	5		yes^a,d^	NA	0	-0.14
EFHD1	5	5		-	NA	0.01	-0.64
TRMT61B	34	0		yes^b,c^	NA	0	0.58
FN1	31	0		yes^c^	Others^2^	0	-0.11
FGG	8	0		-	Other^3^	0	-0.17
PRKAR1A	8	0		yes^a,d,f^	NDD^4^ & Others	0.09	0.07
TROAP	7	1		yes^a,b,c,d^	NA	0	0.32
CYFIP2	6	1		-	NDD (DEE 65)	1	0.10
LZTS2	6	0		yes^a,b,d,f^	NA	0.05	-0.49
CNOT1	5	0		yes^d^	NDD^5^	1	0.81
DHCR7	5	0		-	NDD^6^	0	-0.04
GSPT1	5	0		-	NA	1	0.85
PSMD7	5	0		-	NA	0.26	0.28
SLC25A13	5	0		-	Other^7^	0	0.04
UNC119B	5	0		yes^d^	Other^8^	0.78	0.22
SMC3	6	0		-	NDD^9^		0.70
CDC42BPB	3	8		-	NDD^10^	1	0.07
IGHA1	2	7		-	NA	-	-0.34
NEXN	3	5		-	Other^11^	1	-0.26
RPA1	2	6		-	Other^12^	0	0.31
RNF114	0	19		-	NA^13^	0.05	-0.09
CDC27	0	18		yes^c^	NA	1	0.55
RECQL	0	10		-	Other^14^	0	0.69
ANAPC7	0	8		-	NDD^15^	0.85	0.35
CDC23	0	7		yes^c^	NA	0.99	0.46
AGO1	0	6		-	NDD^16^	1	NA
CDC16	0	6		-	NA	0.61	0.44
PRRC2B	0	6		-	NA	1	0.69
ADAR	0	5		-	NDD^17^	0.61	0.59
ANAPC4	0	5		yes^c^	NA	0	1

This list includes DYRK1A’s protein interactors identified through mass spectrometry analysis. For each protein, the number of peptide iteration found using C-terminal and N-terminal DYRK1A antibodies, information related to their previously described interaction with DYRK1A, their functions, associated disease, gene expression and intolerance to loss-of-function, is provided. *NA*: not applicable; *PSM*: Peptide-Spectrum Matching; *Phospho*: phosphorylation detected for peptides corresponding to DYRK1A itself on Tyr321 but also for FAM117B on Ser106 (ARTpSPTV), FAM53C on SerS324 (RFSLpSPSL), and GLCCI1 on Ser30 (AAGpSPPA) and Ser303 (VEGIpSPEL); *Known partner*: interaction with DYRK1A already described in previous proteomic studies: ^a^Varjosalo et al. (2013); ^b^Li et al. (2015); ^c^Guard et al. (2019); ^d^Menon et al. (2019); ^e^Roewenstrunk et al. (2019); ^f^Huttlin et al. (2021) *Intolerance to LoF*: probability for the gene to be intolerant to loss-of function variants (pLI, from gnomAD); *NDD*: neurodevelopmental disorder; *DEE*: developmental and epileptic encephalopathy; ^1^ DYRK1A syndrome; ^2^ kidney and skeletal diseases; ^3^ blood disease; ^4^ Acrodysostosis; ^5^ Holoprosencephaly & Vissers-Bodmer Syndrome; ^6^ Smith-Lemli-Opitz syndrome; ^7^ Citrullinemia; ^8^ Late-onset cone-rod dystrophy; ^9^ Cornelia de lange syndrome 3; ^10^ Chilton-Okur-Chung syndrome; ^11^ Cardiomyopathy; ^12^ Pulmonary fibrosis & telomeropathy; ^13^ but possible association with psoriasis; ^14^ Bloom Syndrome; ^15^ Ferguson-Bonni syndrome); ^16^ Argonaute syndrome; ^17^ Aicardi-Goutieres syndrome; *Correl. Brain expression*: Pearson’s coefficient measuring the correlation between the spatiotemporal expression of the gene and those of DYRK1A (data from BrainSpan including different 254 brain samples ranging from 8 weeks of pregnancy to 40 years).

We compared the expression of DYRK1A and its partners during brain development across the different brain structures (data from BrainSpan) and found a moderate-to-high positive correlation (Pearson’s coefficient) for one-third of them (11/35; Table 1). Half of DYRK1A’s partners identified in hNSCs (16/35) are known to be involved in human diseases, with neurodevelopmental manifestations for nine of them (PRKAR1A, CYFIP2, CNOT1, DHCR7, SMC3, CDC42BPB, ANAPC7, AGO1, and ADAR; Table 1). Interestingly, of the 73 clinical signs listed in the Human Phenotype Ontology (HPO) as part of DYRK1A syndrome, 80% are also described in diseases caused by mutations in partners of DYRK1A (Supplementary Figure S2). The diseases caused by pathogenic variants in *DHCR7*, *SMC3,* and *CDC42BPB* show the strongest overlap as they share more than 25% of the clinical signs of DYRK1A syndrome (Figure 1C). Among DYRK1A’s newly described partners that are not yet linked to human pathology, some exhibit high intolerance to loss-of-function (LoF) variants (high pLI in the gnomAD database) and present an expression profile during brain development that strongly correlates with DYRK1A. This suggests that these partners could be relevant candidates for NDDs and may contribute to the pathophysiology of DYRK1A syndrome. Notable examples include the well-known partner DCAF7 (pLI = 0.93, Pearson’s correlation = 0.81) as well as GSPT1, a GTP-binding protein involved in translation termination and the regulation of cell cycle progression (1; 0.85).

### *DYRK1A* KD in human neural stem cells affects the expression of genes encoding extracellular matrix proteins

**Figure 2:**
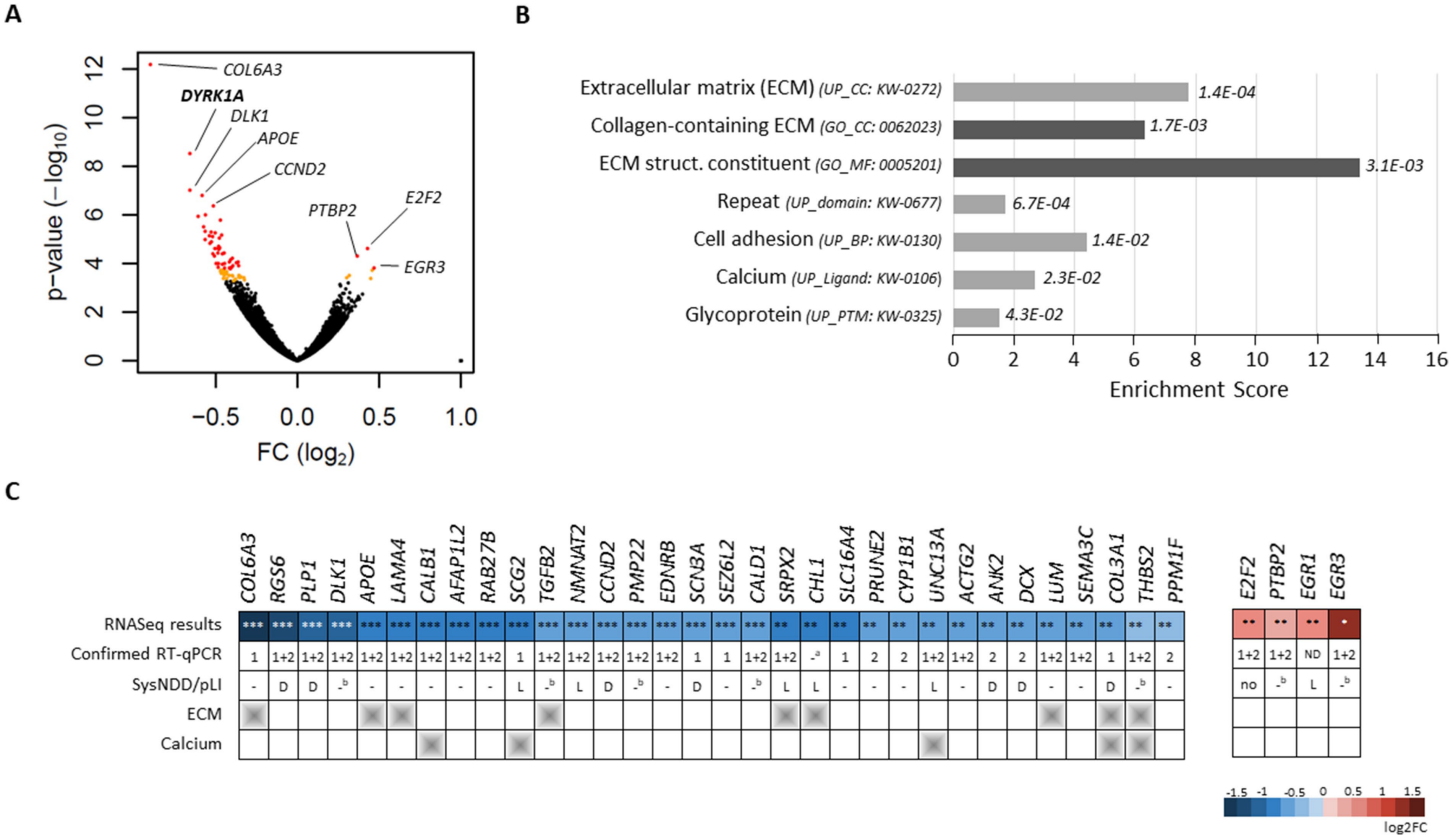
Transcriptomic changes induced by *DYRK1A* knockdown in human neural stem cells. **(A)** Volcano plot showing RNA sequencing data (control *vs.* si*DYRK1A* treated hNSC_1_) with the significance (−log10 of the *p*-value) as a function of the log2 fold change. Genes not significantly deregulated are shown in black; genes significantly deregulated (DEG) with an adjusted p-value between 0.05 and 0.1 are shown in orange, while genes with an adjusted *p*-value <0.05 are shown in red. **(B)** Pathways analysis using Uniprot and Gene Ontology terms revealed significant enrichment in proteins from the extracellular matrix (ECM), involved in cell adhesion and binding calcium; CC, cellular component; MF, molecular function, BP, biological process; PTM, posttranslational modification. **(C)** Representation of RNA-Seq results for a subset of DEG: RNAseq results including adjusted *p*-value (*p-value<0.1, **p-value<0.05, and ***p-value<0.01) and log2 fold change (color intensity), RT-qPCR analysis performed in a third series of hNSC_1_ (*n* = 3) and in three series of hNSC_2_ (*n* = 9; normalized on *GAPDH* and *YWHAZ*); significant change in gene expression confirmed in hNSC_1_ (1), hNSC_2_ (2), or both (1 + 2). Genes involved in neurodevelopmental disorders (NDD) are retrieved from the “Definitive” (*D*) and “Limited” (*L*) gene lists of SysNDD database. ^b^, gene not already associated with an NDD but having a high intolerance to loss-of-function in the gnomAD database (pLI > 0.9).

**Figure 3:**
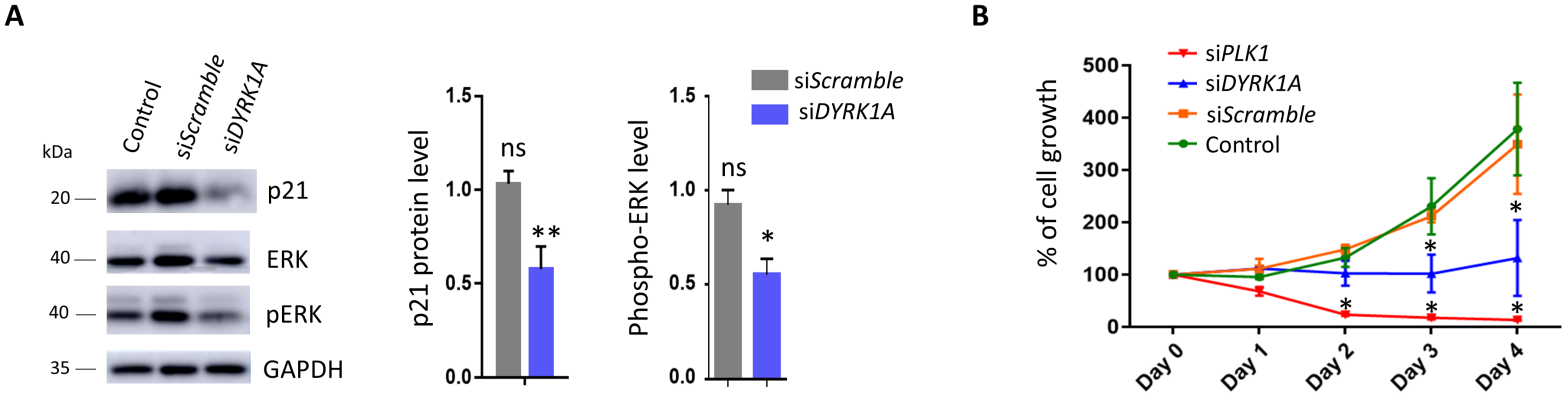
*DYRK1A* knockdown affects human neural stem cell proliferation. **(A)** Western blot analysis on total protein extract from hNSC_1_ treated with lipofectant alone (Control) or transfected with *Scramble* or *DYRK1A* siRNA for 48 h. The level of p21 protein (*n* = 3) and ERK and phosphoERK (pERK; *n* = 3) was normalized to the GAPDH level. Multiple comparison tests were performed using a one-way ANOVA test with Dunnett’s correction: ns: not significant; **: adjusted *p*-value<0.01; *: adjusted *p*-value<0.05; errors bar represent standard error of the mean (SEM); **(B)** proliferation assay performed on hNSC_1_ treated with lipofectant alone (Control), transfected with *Scramble* siRNA, *DYRK1A* siRNA (si*DYRK1A*). A treatment with *PLK1* siRNA (si*PLK1*) was used as a positive control, leading to cell apoptosis. At each time point (days 0, 1, 2, 3, and 4), cells were counted and data normalized with day 0 (*n* = 3 per line). Student’s *t*-test comparison was done comparing to Control condition: *p-value<0.05; error bars represent SEM.

DYRK1A has previously been described as both a direct and indirect transcriptional regulator. Indeed, DYRK1A has been shown to modulate chromatin remodeling complexes and epigenetic marks (Lepagnol-Bestel et al., 2009; Li et al., 2018) and also interacts with various transcription factors such as NFAT (Gwack et al., 2006) and GLI1 (Ehe et al., 2017) and with RNA Pol II (Yu et al., 2019). To identify the effect of *DYRK1A* loss on the regulation of gene expression in hNSCs, we used a pool of siRNAs to knock down (KD) DYRK1A expression in hNSC_1_ (Supplementary Figure S3) and performed mRNA sequencing. This transcriptomic analysis revealed 91 significantly deregulated protein-coding genes (DEG) following *DYRK1A-*KD compared to the control condition, with 85 of these genes being downregulated; DYRK1A was the second most significantly downregulated gene (log2 fold change = −0.87, adjusted *p*-value =1.10E-05; Figure 2A; Supplementary Tables S2, S3). In comparison, no DEGs were found in hNSC_1_ treated with non-specific siRNA (*Scramble*). Uniprot and GO terms analyses revealed an enrichment of genes related to extracellular matrix (ECM) components and the plasma membrane (e.g., *COL6A1, COL6A3, LUM*, and *THBS2*), as well as calcium binding (e.g., *CALB1*, *SGC2*, and *UNC13A*) among the DEGs (Figure 2B). Only six genes (*EGR1*, *EGR3*, *E2F2*, *HMRB2* and *TYMS*) were found to be significantly upregulated. Among them, two were members of the early growth factor (EGR) transcription factors families, *EGR1* and *EGR3*. We then checked the expression of the most significant DEGs using multiplex RT-qPCR in a new series of the same hNSC line (hNSC_1_) and three series of an additional hNSC line from another genetic background (hNSC_2_) treated by *DYRK1A* siRNA. We confirmed significant changes in gene expression in both cell lines for 23 genes (Figure 2C). No significant motif enrichment was found in the promoter region (−1,000/+100 bp) of the DEGs using the Analysis of Motif Enrichment, version 5.1.1 (AME) tool. Among the DEGs, strong evidence of involvement in neurodevelopmental disorders was reported for 15 of them (“definitive” gene list from SysNDD, e.g., *PLP1, RGS6*, and *SCN3A*) and moderate evidence for 10 more (“limited” gene list from SysNDD, e.g., *UNC13A, NMNAT2, and CHL1*). Some of the DEG are not yet associated with human disease but are highly intolerant to LoF. Among them, *PTBP2* has a high pLI and exhibits a strong expression correlation with *DYRK1A* during brain development (Pearson’s correlation = 0.8), suggesting that it could be a novel candidate gene for NDDs. Interestingly, if an increase of *PTPB2* mRNA was observed after *DYRK1A* KD in both hNSC lines, a significant decrease in protein level was observed (Supplementary Figure S4). The same observation was made for the gene *CDKN1A*, which encodes the p21 protein, as a decrease in protein level was detected after *DYRK1A* KD, while an increase of mRNA was detected using Leafcutter, a program designed to detect changes in exon/intron ratios (Supplementary Table S5; Figure 3A; Supplementary Figure S5), affecting a weakly expressed non-canonical isoform with unknown function (NM_001291549.1). An overexpression of *E2F2* mRNA was observed, which prompted us to analyze the expression of all known target genes of the E2F signaling pathway. Among the 200 genes belonging to this molecular signature, 91% were overexpressed in the *DYRK1A-*KD condition (Supplementary Figure S6), suggesting incomplete repression of E2F-mediated gene transcription, similar to what has been reported in human lymphocytes (Thompson et al., 2015). In contrast, we checked the expression of TGFB target genes as both *TGFB2* and *TGFBI* mRNA were found to significantly decrease after *DYRK1A*-KD and observed that they are mainly downregulated (42/54 with a log2FC <0), highlighting repression of the TGF*β* pathway after *DYRK1A* loss.

### DYRK1A inactivation reduces human neural stem cell proliferation and ERK activation

The interaction of DYRK1A with APC members and other proteins involved in cell cycle regulation, as well as the changes in expression of p21, E2F, and others identified after *DYRK1A-*KD suggest a role of DYRK1A in the proliferation of hNSCs, consistent with what was observed in other cell types (Park et al., 2010). Therefore, we analyzed the ability of hNSCs to proliferate after *DYRK1A*-KD and observed a significant decrease in cell proliferation 72 h following DYRK1A-KD in hNSCs, compared to a control condition or *Scramble* siRNA (Figure 3B; Supplementary Figure S7A). Because of the implication of p21/CDKN1A in cellular senescence, we tested whether DYRK1A-KD affects the number of cells undergoing senescence using the SA-β galactosidase assay 4 days post-transfection. Counting revealed that few cells turned positive after SA-β galactosidase labeling and that neither si*Scramble* nor si*DYRK1A* treatments appeared to increase the number of senescent cells (Supplementary Figure S8). As one of the novel DYRK1A partners we identified, RNF114 is a ubiquitin ligase that regulates cell cycle progression by degrading p21 (Han et al., 2013). We tested the consequences of its KD in hNSCs (Supplementary Figure S9A). However, if a decrease in p21 protein levels was observed after *RNF114-*KD (Supplementary Figure S9B), no obvious effect on proliferation was observed (Supplementary Figure S8C), suggesting that the decrease in hNSC proliferation caused by *DYRK1A*-KD is not mediated by its interaction with RNF114. Recently, a study conducted in a conditional *Dyrk1a* heterozygous mouse model identified several alterations in growth signaling cascades, such as ERK and mTOR (Levy et al., 2021). Interestingly, a significant decrease in ERK phosphorylation was observed in hNSCs 48 h after treatment with si*DYRK1A* (Figure 3A).

## Discussion

Our study contributes to a growing understanding of the molecular role of DYRK1A in human neural development, using the model of human neural stem cells (hNSCs), by characterizing its interactome, its impact on gene expression, and its regulatory functions on cellular processes such as proliferation. The findings shed light on how DYRK1A interacts with proteins or regulates the expression of genes known to be involved in neurodevelopmental disorders (NDDs). Additionally, they highlight other genes that, due to their intolerance to loss-of-function and expression profiles during brain development, emerge as strong candidates for involvement in NDDs.

Through immunoprecipitation and mass spectrometry (IP-MS), we identified 35 DYRK1A interactors in human neural stem cells, with an important overlap with those previously identified in other cell types (15/35), highlighting a strong conservation of DYRK1A interactions across different tissues. These findings show that DYRK1A engages with multiple proteins involved in cell cycle regulation, transcriptional control, and signaling pathways critical to neurodevelopment. At the intersection of these different biological processes lies the large E3 ubiquitin ligase machinery of the anaphase-promoting complex (APC; Pal and Summers, 2017; Skaar and Pagano, 2009), with five of its members (ANAPC3, 4, 6, 7, and 8) identified as interacting with DYRK1A. Recently, another study also highlighted DYRK1A interaction with three APC factors (ANAPC3/CDC27, ANAPC4, and ANAPC8/CDC23; Guard et al., 2019), which we validated in this study. Interestingly, four of these core subunits (ANAPC3-6-7 and 8) compose the tetratricopeptide repeat (TPR) lobe APC subcomplex, playing a role in the global scaffolding of the complex and substrate recognition (Sivakumar and Gorbsky, 2015). This would suggest that DYRK1A may interact with APC through the TPR lobe and given the kinase activity of DYRK1A, could possibly phosphorylate the subunit and therefore play a role in the global activity of the complex. The pronounced enrichment of ANAPC3/CDC27 suggests it may be the primary interaction partner of DYRK1A. Another ubiquitin E3 ligase, RNF114/ZNF313, playing a role in the regulation of cell cycle and repression of senescence, was detected in our study. Interestingly, although this gene is not directly involved in NDD, a polymorphism affecting its expression is associated with an increased risk of developing psoriasis (Capon et al., 2008). Notably, we recently showed that inflammatory skin conditions are frequent in individuals with DYRK1A syndrome (Courraud et al., 2021). RNF114 ubiquitinates p21/CDKN1A, leading to its degradation (Han et al., 2013). Surprisingly, its knockdown in hNSCs leads to a decrease in p21 protein levels, like what is observed with *DYRK1A* knockdown, while, unlike *DYRK1A* knockdown, it has no effect on the proliferation of hNSCs. These results indicate that the downregulation of RNF114 and p21 proteins alone is not sufficient to cause the decreased proliferation identified in the *DYRK1A*-KD condition.

Importantly, several DYRK1A interactors identified in our study exhibit significant expression correlation with DYRK1A during brain development. Some are mutated in NDDs, supporting their relevance in neurodevelopmental pathways. The dysfunction of these interacting proteins may contribute to the shared clinical manifestations of DYRK1A-related disorders (such as short stature, microcephaly, strabismus, and scoliosis). Others are not associated yet with any human disease but have a high intolerance to loss-of-function (LoF) variants, which warrants further investigation for their potential implication in NDDs. It is the case for instance for GSPT1 or DCAF7, for which de novo missense variants classified as “probably deleterious” have been identified in individuals with NDDs (Supplementary Table S5). The identification of additional patients will be helpful in confirming these genes as novel NDD genes.

We identified genes differentially expressed in hNSCs after a transient *DYRK1A* knockdown (KD) showing little overlap with previous transcriptomic studies conducted in murine B and T cells (Thompson et al., 2015), zebrafish brain (Cho et al., 2019), xenopus brain (Willsey et al., 2020), and human HeLa cells (Vona et al., 2015). In hNSCs, *DYRK1A-*KD resulted in the dysregulation of genes encoding proteins associated with the extracellular matrix (ECM) and calcium-binding proteins. Many of these calcium-binding ECM proteins, such as COL3A1 and THBS2, play roles in cellular adhesion and structure, potentially impacting neural cell interactions and therefore the balance between proliferation and differentiation during brain development. Interestingly, mutations in these two genes are responsible for a disorder of connective tissues, Ehlers-Danlos syndrome, a connective tissue disorder characterized by fragile skin, which is also frequently observed in DYRK1A syndrome.

Only a few genes were found significantly upregulated after *DYRK1A-*KD. Among them, *PTBP2,* an RNA-binding protein, regulates alternative splicing, polyadenylation, mRNA stability, and translation and plays a role in neural development by regulating alternative splicing in brain tissues (Hu et al., 2018). Interestingly, despite the significant upregulation of *PTBP2* mRNA following *DYRK1A*-KD, we observed a marked decrease in PTBP2 protein expression. Similar results were obtained for *CDKN1A*, where the mRNA level of a long minor isoform (potentially encoding a protein that is 34 amino acids longer than the main isoform, with an unknown function) increased after *DYRK1A-*KD, while the amount of P21/CDKN1A protein was significantly reduced. These findings suggest that *DYRK1A-*KD may lead to decreased protein stability or degradation, triggering compensatory mechanisms that result in increased transcription of the corresponding mRNA.

On the one hand, DYRK1A interacts with proteins and complexes involved in cell proliferation regulation, such as APC; conversely, *DYRK1A*-KD impacts several signaling pathways critical for cell proliferation. These include increased expression of the transcription factor *E2F2* and their target genes, elevated expression of *EGR* genes, reduced expression of the growth factor *TGFβ,* decreased levels of the p21 protein, and diminished ERK activation. Although these effects on distinct growth signaling pathways appear contradictory, we demonstrated that the overall impact of *DYRK1A*-KD is a significant reduction in hNSC proliferation. The majority of previous published studies report decreased proliferation associated with *DYRK1A*-KD, as observed in *Drosophila* (Tejedor and Hämmerle, 2011) or in mouse models (Laguna et al., 2013; Guedj et al., 2012; Barallobre et al., 2014). DYRK1A’s role in neuronal precursor proliferation has been recognized since its discovery in the Drosophila mnb model (Fischbach and Heisenberg, 1981), where reduced cell numbers and brain size were observed in mutant flies. While DYRK1A has been identified as both a positive and negative regulator of cellular proliferation depending on the context, reduced DYRK1A expression generally results in decreased cell proliferation across various models. For example, heterozygous KO *Dyrk1a* +/− drosophila, mice, zebrafish, or xenopus showed reduced brain size (Fotaki et al., 2002; Willsey et al., 2020; Tejedor et al., 1995; Kim et al., 2017) attributed to decreased neuronal precursor proliferation during neurogenesis. The effect of DYRK1A inhibition on hNSC proliferation had not been explored prior to our study. However, Bellmaine et al. highlighted that DYRK1A inhibition disrupts neural specification in human embryonic stem cells (ESCs; Bellmaine et al., 2017). Future studies employing non-transient inactivation of *DYRK1A*, such as hNSCs differentiated from induced pluripotent stem cells with a loss-of-function mutation in *DYRK1A*, could provide further insights into how DYRK1A loss affects hNSC proliferation (cell cycle elongation, increase of apoptosis, etc.) and differentiation.

In conclusion, this study enhances the understanding of DYRK1A’s multifaceted role in human neurodevelopment. By characterizing DYRK1A’s interactome in human neural stem cells, its effects on gene expression, and its functional impact on proliferation, we reveal critical insights into its role in brain development and the molecular basis of associated neurodevelopmental disorder. Future research could explore therapeutic modulation of DYRK1A activity or its interactors, providing new avenues for intervention in DYRK1A syndrome. Our findings emphasize the importance of balanced DYRK1A expression in neurodevelopment and support its continuing study as a central player in the complex network underlying cognitive development and function.

## Data Availability

The datasets presented in this study can be found in online repositories Gene Expression Omnibus: GSE247739. The names of the repository/repositories and accession number(s) can be found in the article/Supplementary material.

## Footnotes

^1^ https://bioserv.rpbs.univ-paris-diderot.fr/services/Proteo3Dnet/

^2^ https://www.gsea-msigdb.org/gsea/msigdb/
