# Correction: DYRK1A roles in human neural progenitors

**DOI:** 10.3389/fnins.2026.1786289

**Published:** 2026-02-16

**Authors:** Jeremie Courraud, Angélique Quartier, Nathalie Drouot, Irene Zapata-Bodalo, Johan Gilet, Alexandra Benchoua, Jean-Louis Mandel, Amélie Piton

**Affiliations:** 1 Institut de Génétique et de Biologie Moléculaire et Cellulaire, Illkirch, France; 2 Centre National de la Recherche Scientifique, UMR7104, Illkirch, France; 3 Institut National de la Santé et de la Recherche Médicale, U964, Illkirch, France; 4 Strasbourg University, Illkirch, France; 5 I-Stem, Evry, France; 6 Genetic Diagnosis Laboratory, Strasbourg University Hospital, Strasbourg, France; 7 Institut Universitaire de France, Paris, France

**Keywords:** DYRK1A, human neural stem cells, interactome, gene expression, cell proliferation, RNF114, p21/CDKN1A, neurodevelopmental disorders

There was a mistake in Figure 1 as published. The name of one protein was misspelled, the interactor **GSPT1** was misspelled “*GSTP1*”, which referred to a different protein.

The corrected Figure 1 appears below.

**Figure 1:**
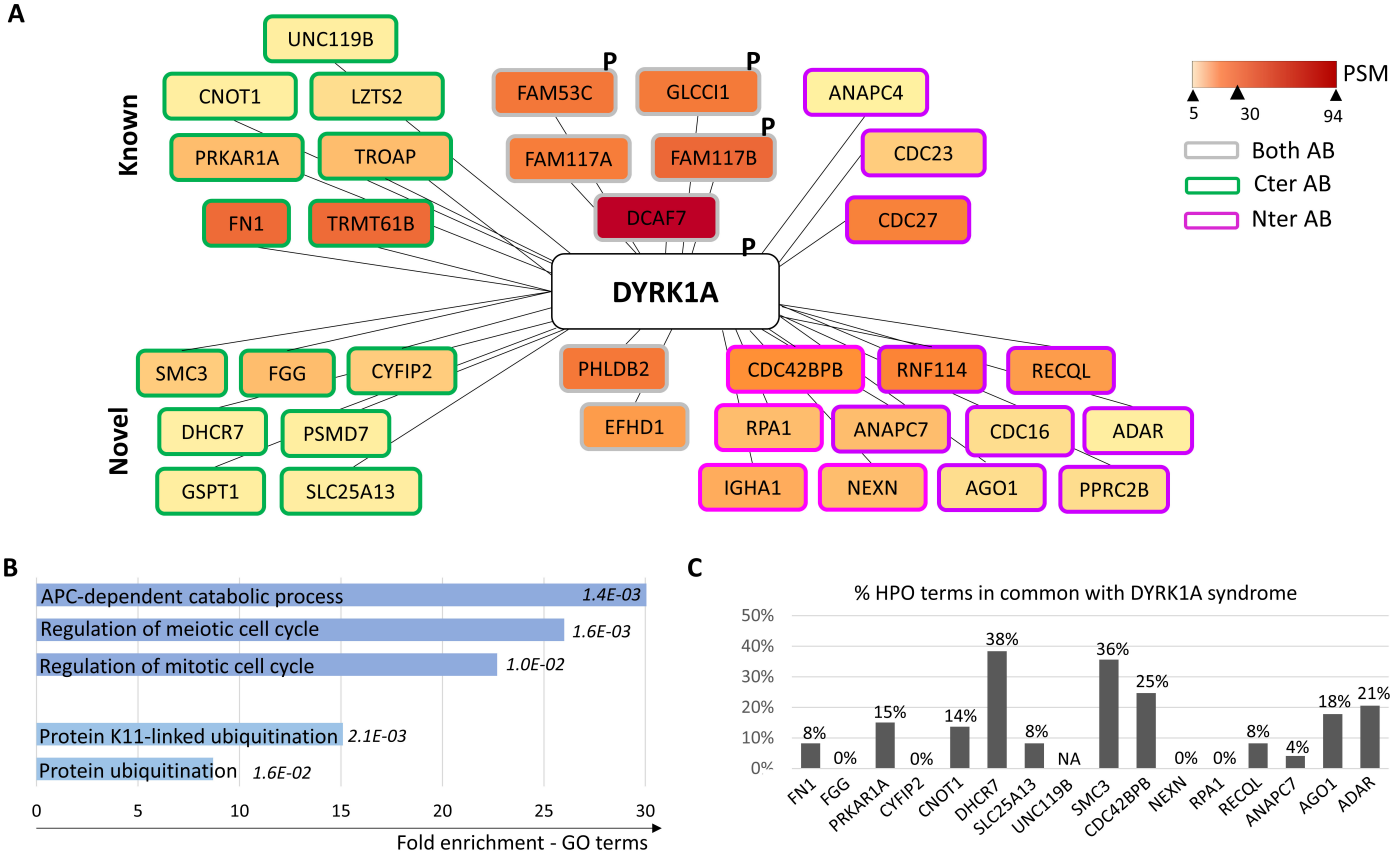
Representation of DYRK1A protein interactome in human neural stem cells. **(A)** Protein interactome of DYRK1A: boxes are filled with a yellow to dark red gradient corresponding to the number of peptide-spectrum matching (PSM) values (see Supplementary Table S1); proteins found using the anti-DYRK1A antibody directed against the C-terminal part of the protein are circled in green, those found using the one directed against the N-terminal part in pink, and those found with both antibodies in gray; “P” indicates that phosphorylation events have been identified. **(B)** Significant enrichment in GO terms among DYRK1A interactors. **(C)** Number of Human Phenotype Ontology (HPO) terms of DYRK1A syndrome reported in the diseases associated with DYRK1A's partners.

The original version of this article has been updated.

