# Supplementary material for: DYRK1A roles in human neural progenitors
Source: Front Neurosci. 2025 Mar 13;19:1533253. doi: 10.3389/fnins.2025.1533253 (PMC11966461; doi:10.3389/fnins.2025.1533253)
Supplement: Supplementary file 2 [file Data_Sheet_1.docx]

**Figure S1. Representation of functional networks involving DYRK1A partners identified in hNSC**

The network analysis was performed using Proteo3Dnet (<https://bioserv.rpbs.univ-paris-diderot.fr/services/Proteo3Dnet/>) on the list of 35 interactors identified (Table 1). Four major biological processes are observed, including Anapahase Promoting complex (APC), DNA repair, signaling pataways and regulation of transcription/mRNA regulation; DYR1A= DYRK1A; RECQ1=RECQL; GLCI1=GLCCI1; ERF3A=GSTP1; F117A/B=FAM117A/B; MRCKB=CDC42BPB; KAP0=PRKAR1A; FINC=FN1; S2513=SLC25A13; RPB1=POLR2A; TR61B=TRMT61B.

**Figure S2. Representation of the Human Phenotype Ontology (HPO) terms associated to DYRK1A syndrome found in diseases caused by mutations in DYRK1A partners**

**Figure S3. *DYRK1A* KD leads to decrease of its mRNA and protein levels**

**(A)** RT-qPCR analysis of DYRK1A mRNA level (normalized on GAPDH and YWHAZ) performed on both hNSC lines (hNSC_1_ and hNSC_2_) transfected with Scramble siRNA (siScramble) or DYRK1A siRNA (siDYRK1A) and compared to the condition with transfectant alone (Control) (n=3 per line); **(B)** Western Blot analysis on total protein extract from hNSC_1_ treated with lipofectant alone (Control) or transfected with Scramble or *DYRK1A* siRNA. The quantification of DYRK1A protein level was performed (n=3 per line) with normalization on GAPDH level. Multiple comparisons tests were performed using one-way ANOVA test with Dunnet’s correction: ns: not significant; ***, adjusted p-value<0.001; Error bars represent SEM (standard error of the mean).

**Figure S4. *DYRK1A* KD increases PTBP2 mRNA but decrease its protein level**

Western Blot analysis on total protein extracts from hNSC_1_ and hNSC_2_ treated with lipofectant alone (Control) or transfected with Scramble or *DYRK1A* siRNA. The quantification of PTBP2 protein level was performed (n=3 per line) with normalization on GAPDH level. Multiple comparisons tests were performed using one-way ANOVA test with Dunnet’s correction: ns: not significant; *, adjusted p-value<0.05; Error bars represent SEM (standard error of the mean).

**Figure S5. *DYRK1A* KD increases a non-canonical isoform of *CDKN1A* gene**

**(A)** IGV visualization of RNAseq data from hNSC_1_ for the control, si*Scramble* and si*DYRK1A* conditions for the *CDKN1A* locus, representing the canonical NM_000389.5 isoform (iso1) as well as the alternative NM_001291549.1 isoform (iso2). The red frame indicates the events detected as significantly increased in *DYRK1A* siRNA condition using LeafCutter program; qPCR primers (F1, F2 and R) used for RT-qPCR validation are represented by black arrows. **(B)** RT-qPCR analysis of *CDKN1A* isoform NM_000389.5 (iso1) and NM_001291549.1 isoform (iso2) (normalized on *GAPDH* and *YWHAZ*) in both hNCs lines (hNSC_1_ and hNSC_2_) after transient knock-down (KD) of *DYRK1A* (n=3 for hNSC_1_ and n=9 for hNSC_2_). Multiple comparisons tests were performed using one-way ANOVA test with Dunnet’s correction: ns: not significant; ***: p-value <0.001; Errors bars represent SEM (standard error of the mean).

**Figure S6. Volcano plot representing changes in gene expression of E2F and TGFB1 targets**

Gene lists corresponding to the 200 E2F-targets (n=200) and TGFB1-upregulated targets (n=54) were retrieved through Human Molecular Signatures Database (MSigDB https://www.gsea-msigdb.org/gsea/msigdb/). RNAseq results (control *vs.* si*DYRK1A* treated hNSC_1_) for these genes are represented as a volcano plot showing the significance (-log10 of the p-value) as a function of the log2 fold change (FC).

**Figure S7. Effect of *DYRK1A* knock-down on hNSC_2_ proliferation**

**(A)** Proliferation assay performed on hNSC_2_ line treated with lipofectant alone (Control), transfected with Scramble siRNA (si*Scramble*), *DYRK1A* siRNA (si*DYRK1A*). A treatment with *PLK1* siRNA (si*PLK1*) was used as a positive control, leading to cell growth arrest. At each time point (day 0, 1, 2, 3, and 4), cells were counted and data normalized with day 0 (n=3 per line). Student’s t test comparison was done comparing to Control condition: *: p-value<0.05; Errors bars represent SEM. **(B)** Western Blot analysis on total protein extract from hNSC_2_ treated with lipofectant alone (Control) or transfected with Scramble or *DYRK1A* siRNA for 48h. The level of p21 protein (n=3 per line) was normalized by GAPDH level. Multiple comparison tests were performed using one-way ANOVA test with Dunnet’s correction: ns: not significant; **: adjusted p-value<0.01; *: adjusted p-value<0.05; Errors bars represent SEM (standard error of the mean);

**Figure S8. *DYRK1A* KD does not affect senescence in hNSC.**

**(A)** Evaluation of *DYRK1A* KD effect on senescence induction by quantification of SA-β galactosidase positive cells in both hNSC lines (hNSC_1_ and hNSC_2_) treated with lipofectant alone (Control) or transfected with *Scramble* siRNA (siScramble); *DYRK1A* siRNA (siDYRK1A). Multiple comparisons tests were performed using one-way ANOVA test with Dunnet’s correction: ns: not significant; Errors bars represent SEM (standard error of the mean); **(B)** Representative pictures of hNSC_1_ and hNSC_2_ after 8Gy irradiation treatment (irradiated) or treated by with lipofectant alone (Control) or transfected with *Scramble* or *DYRK1A* siRNA for 48h and labeled by SA-β galactosidase assay. Cells were fixed 4 days after irradiation or transfection. Scales bars (in black) represent 50μm.

**Figure S9. Absence of effect of *RNF114* knock-down on hNSC proliferation**

**(A)** Western Blot performed on hNSC_2_ protein extracts immunoprecipitated with beads only (beads), antibody rabbit anti-mouse not directed against human protein (RAM) and antibody directed against the N-terminal part of DYRK1A protein (DYRK1A Nter). Detection was performed with DYRK1A and RNF114 antibodies **(B)** Western Blot performed on both hNSC lines treated with lipofectant only (control), siRNA scramble or siRNA against *DYRK1A* and revealed using anti-RNAF114 and anti-p21 antibodies **(C)** Proliferation assay performed on both hNSC lines (hNSC_1_ and hNSC_2_) treated with (Control) or without (Control w/o transfectant) lipofectant alone or transfected with Scramble, *DYRK1A*, *RNF114*, *DYRK1A* plus *RNF114*, or *PLK1* siRNA. Cells were counted at day 2 and day 4 and data normalized by the control condition. Statistical analysis was performed using one-way ANOVA Kruskal–Wallis test with Dunnet’s correction for multiple testings: *, p-value<0.05; Errors bars represent SEM.

**Table S1. Peptides detected by Mass Spectrometry from hNSC protein extracts after immunoprecipitation using antibodies against the N-terminal or C-terminal part of DYRKA.** *PSM*, peptide-spectrum match; *AcMAR* : mouse anti-rabbit control antibody, *AcRAM* : rabbit anti-mouse antibody ; *XIC*, extracted ion chromatogram

**Table S2. List of genes DE in *DYRK1A* KD in human neural stem cells (hNSC)**

This list includes genes differentially expressed (adjusted p-value<0.01) after *DYRK1A* KD in hNSC_1_. Associated to these genes, the information related to RNA sequencing: rank classed by adjusted p-values in RNAseq analysis, log2 fold change (*log2FC*), p-value and adjusted p-value but also data related to RT-qPCR analysis in hNSC_1_ and hNSC_2_ lines with their associated p-value calculated using Student test. The implication of each gene in NDD was retrieved from SysNDD (https://sysndd.dbmr.unibe.ch/), and included genes clearly involved in NDD (*definitive*) and candidate genes (*limited*); *Express. Corr BS*, Correlation coefficient between the expression of the gene and that of *DYRK1A* in different brain tissues over time (between 8 weeks of pregnancy and 40 years, BrainSpan data, 254 brain samples); *Intolerance to LoF*, probability for the gene to be intolerant to loss-of function variants (pLI, from gnomAD [0 to 1])

**Table S3. Gene expression changes detected by DESeq on RNAsequencing data after DYRK1A knock-down**

This list includes raw information of the DE genes identified through RNA sequencing in KD

hNSC_1_. Associated to each gene its normalized number of reads, log2 Fold Change, p-values

and adjusted p-values of si*DYRK1A* condition compared to Control.

**Table S4. Change in exon specific expression detected by LeafCutter on RNAsequencing data after DYRK1A knock-down**

**Table S5. List of potential deleterious de novo variants reported in individuals with NDD in *DCAF7* and *GSPT1* (data retrieved from Decipher)**

**
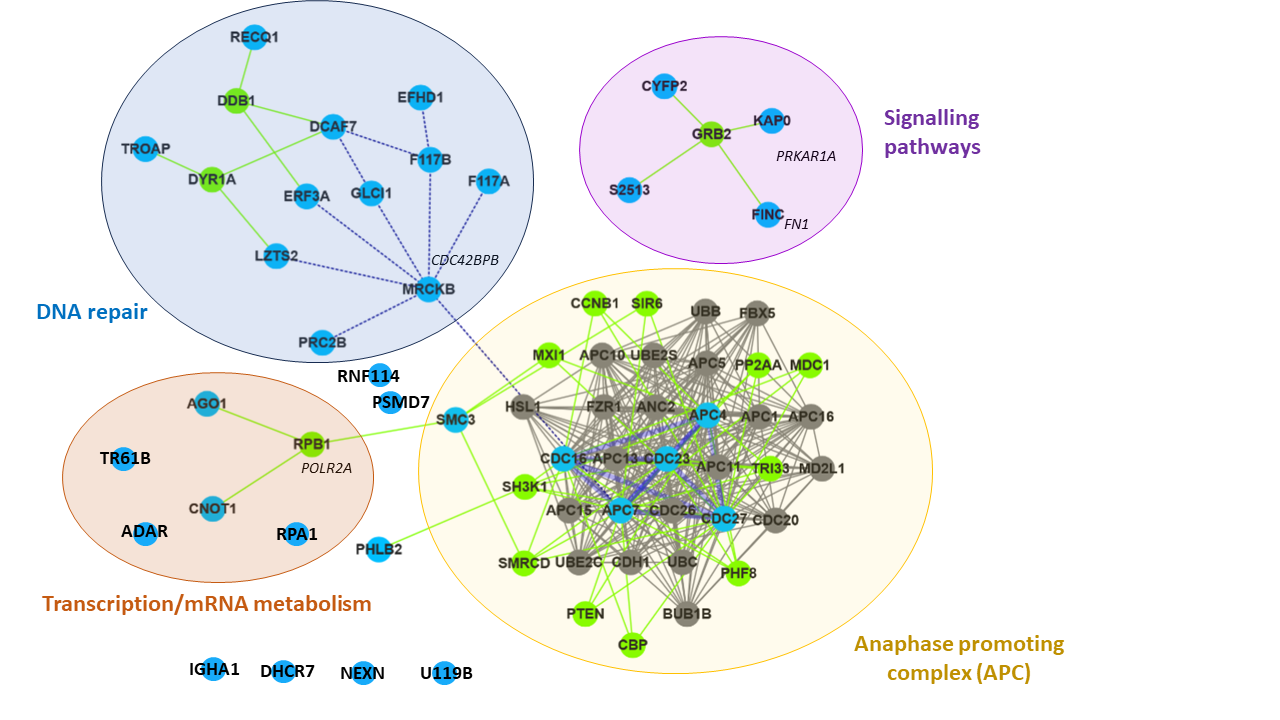
**

Figure S1

**
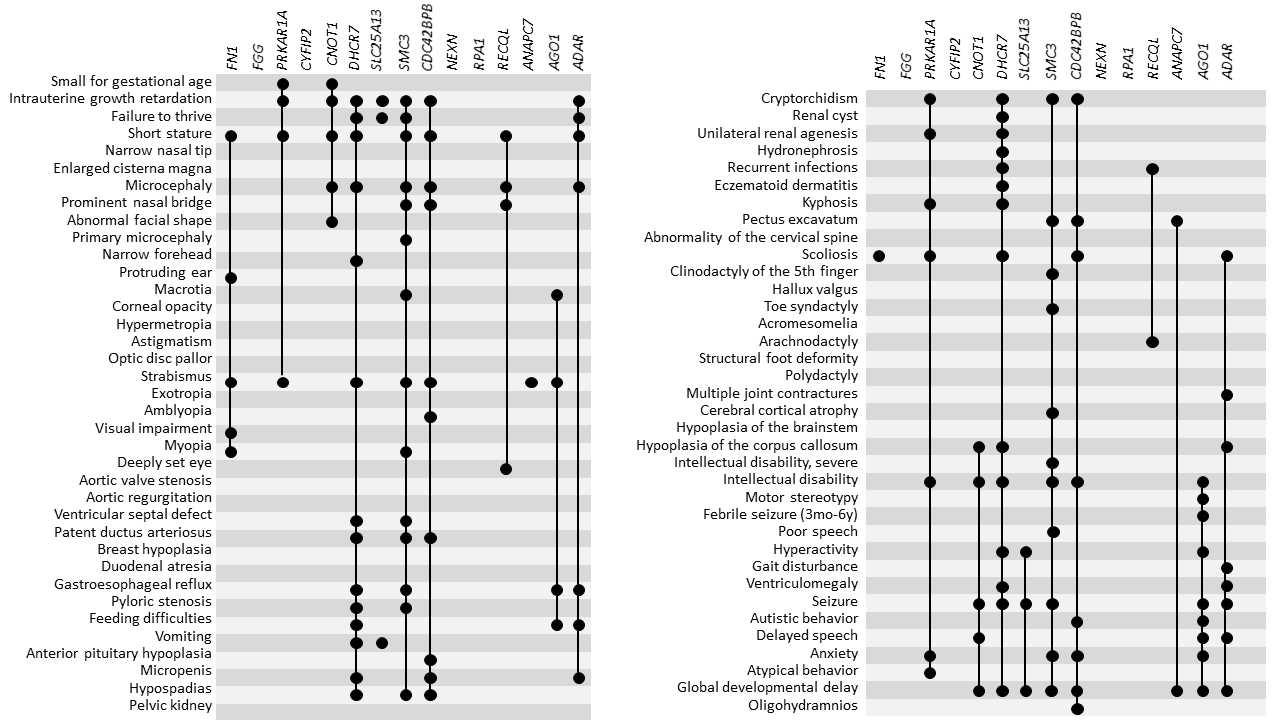
**

**
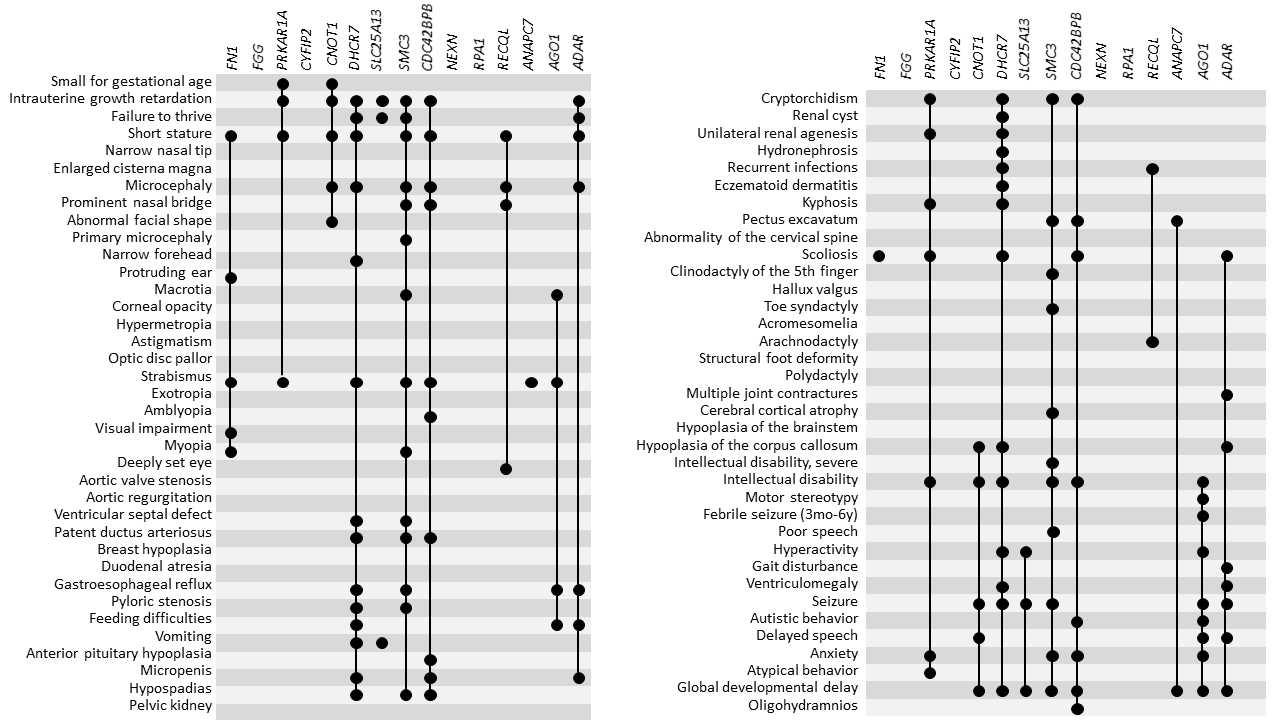
**

Figure S2


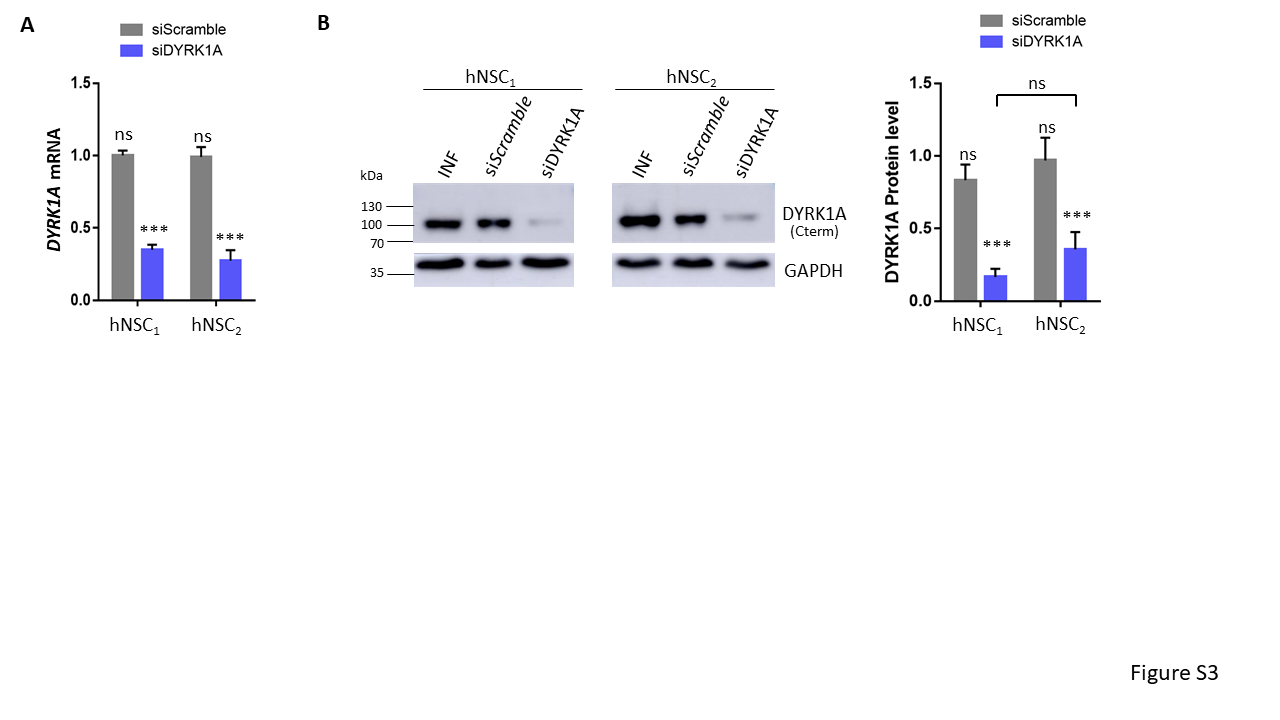


Figure S3

**
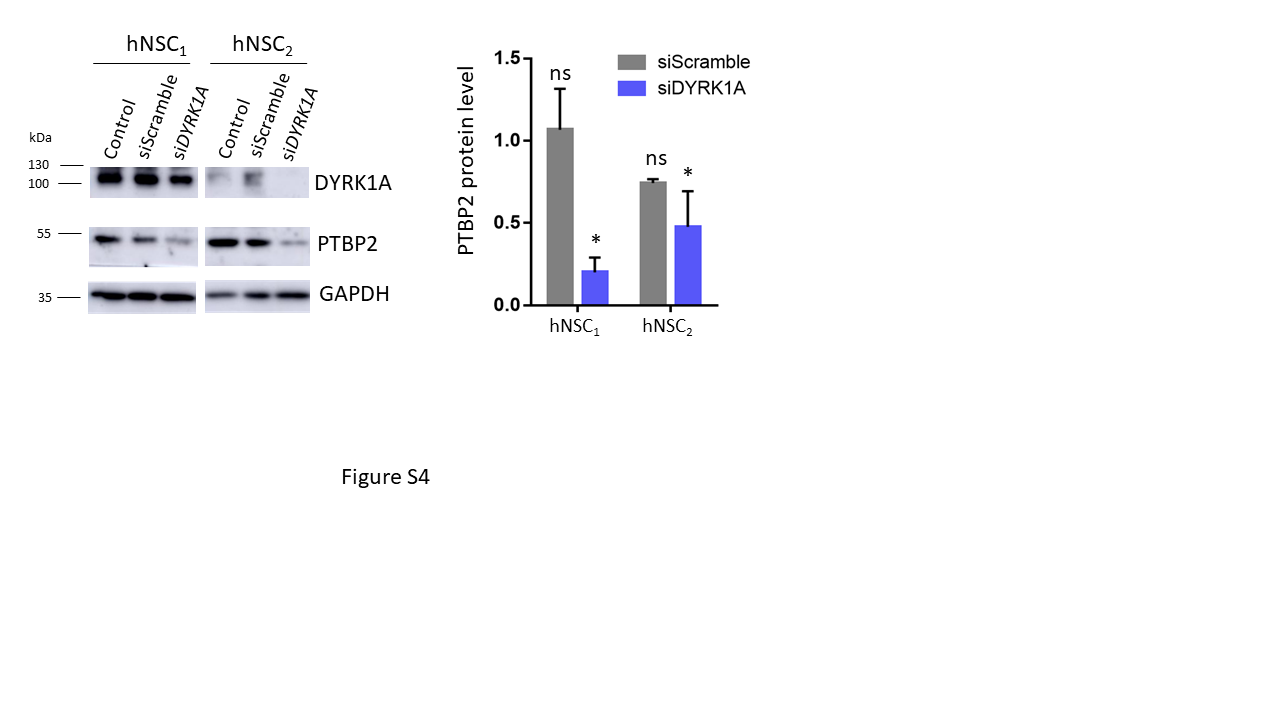
**

Figure S4


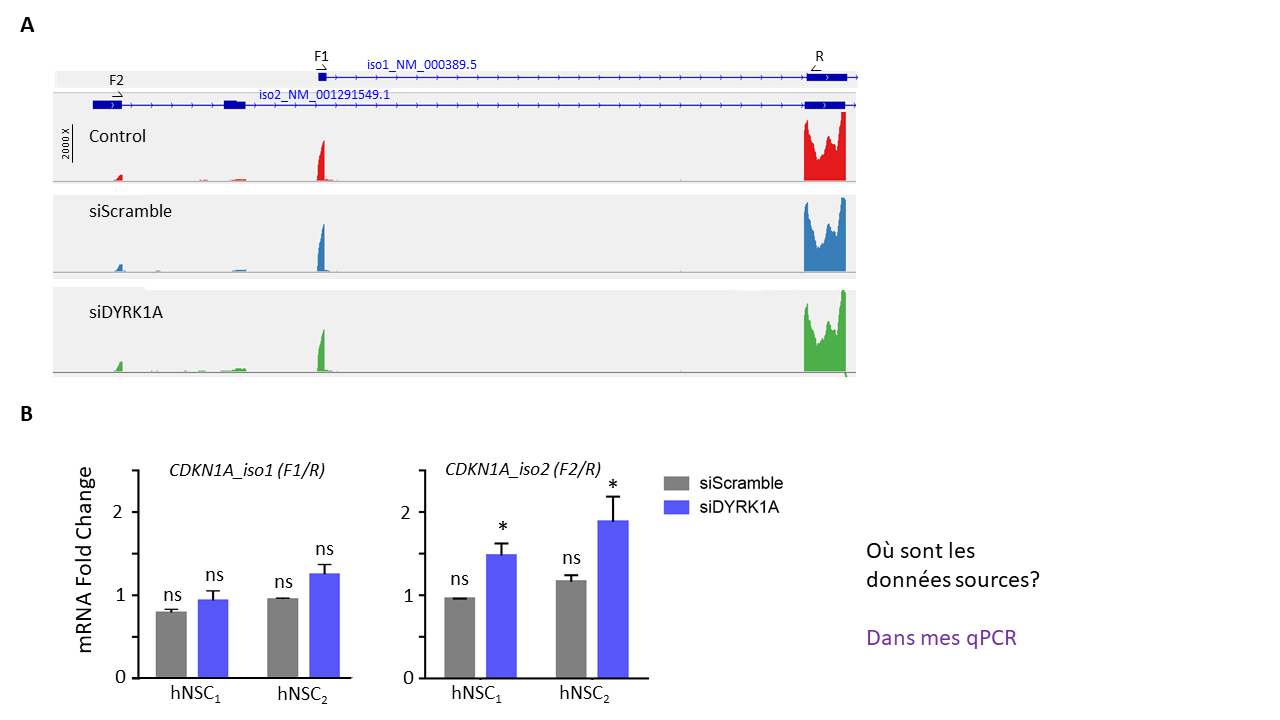


Figure S5


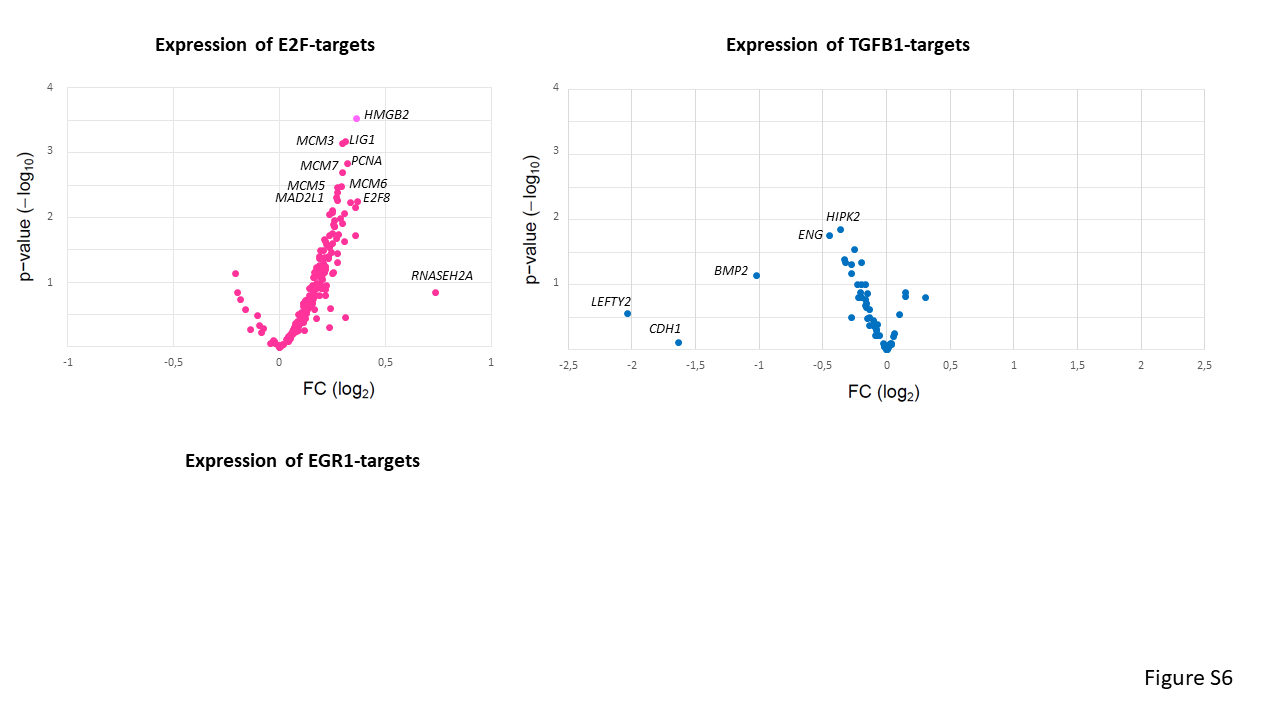


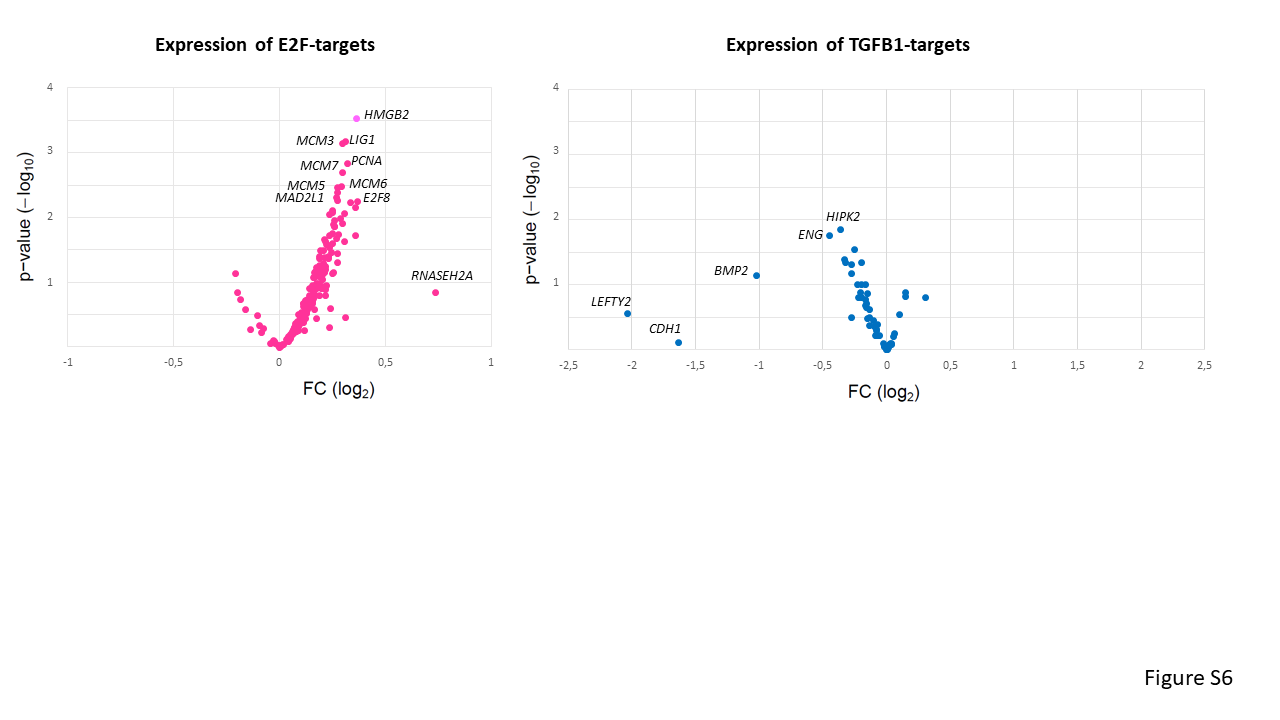


Figure S6


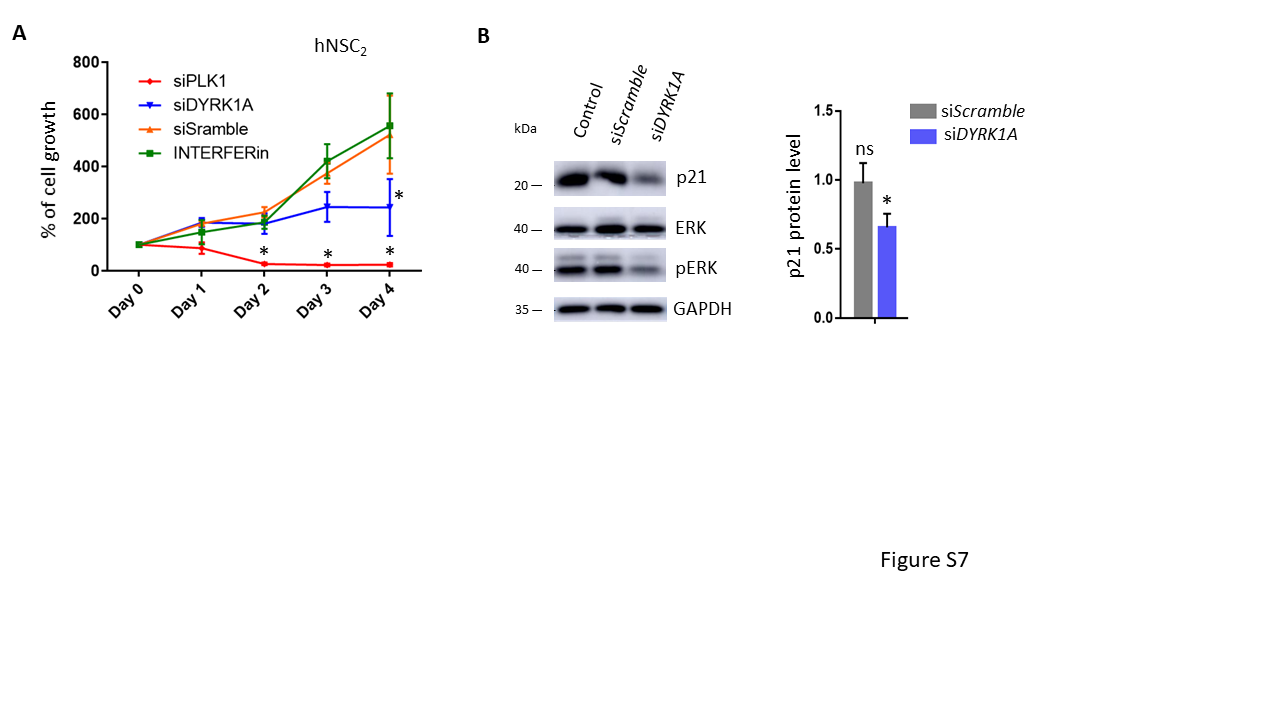

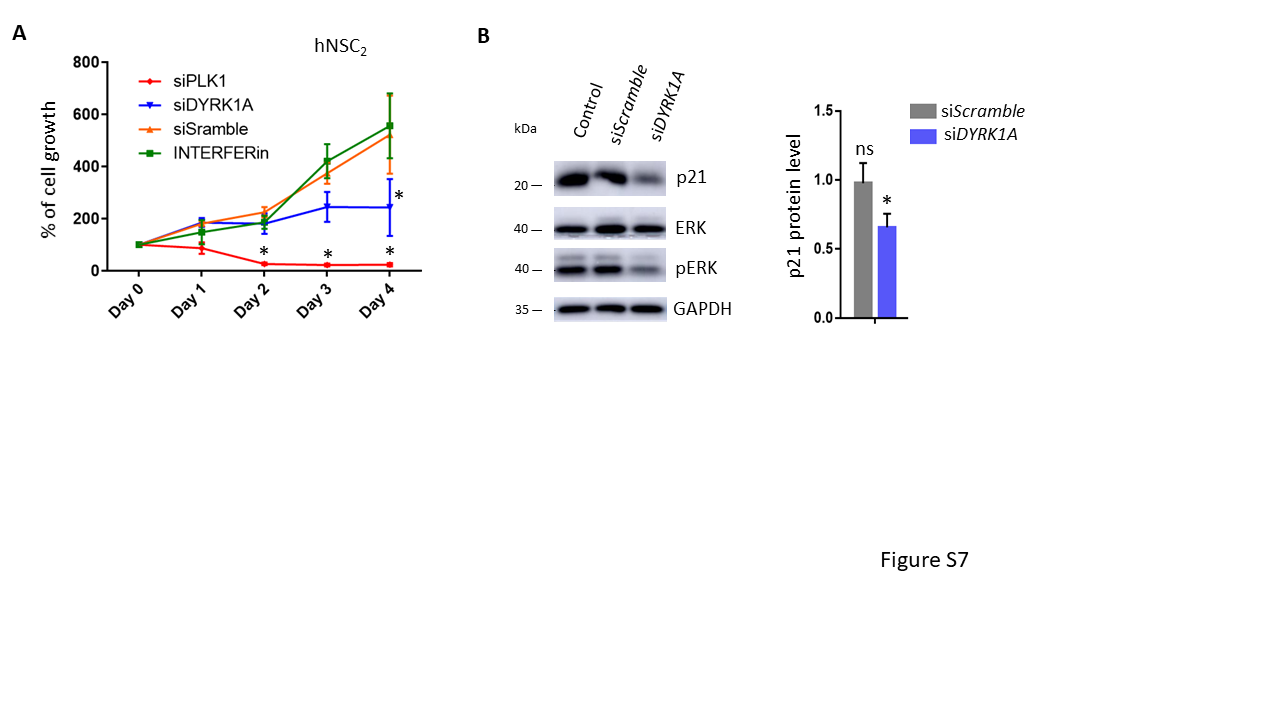


Figure S7


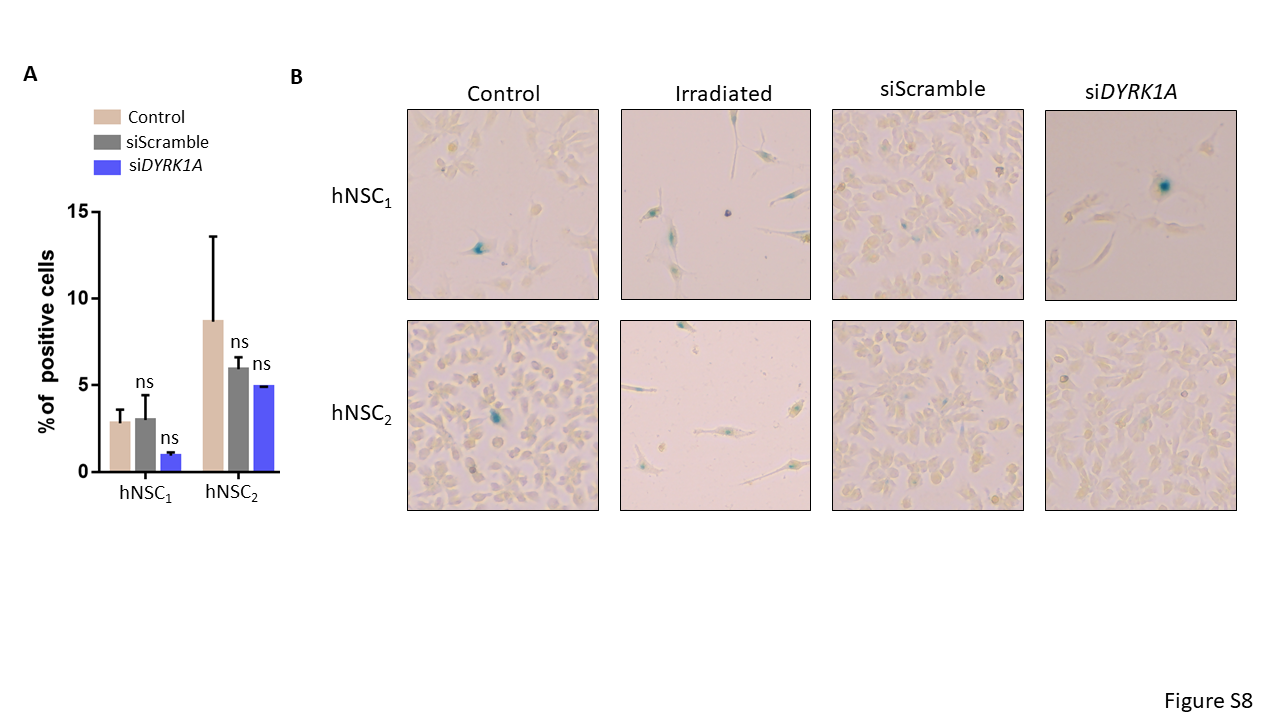


Figure S8


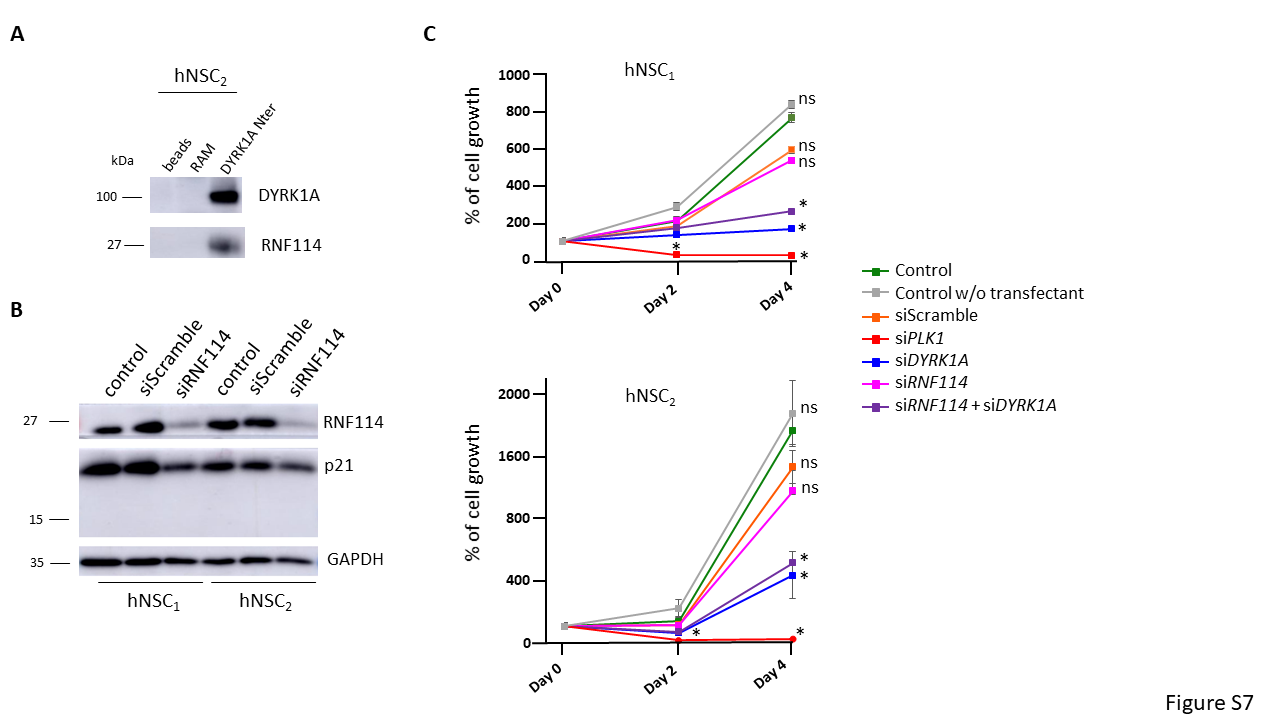


Figure S9
